# Anti-Thyroid Peroxidase Antibodies and Male Gender Are Associated with Diabetes Occurrence in Patients with Beta-Thalassemia Major

**DOI:** 10.1155/2016/1401829

**Published:** 2016-03-31

**Authors:** Giovanni M. Pes, Francesco Tolu, Maria P. Dore

**Affiliations:** ^1^Department of Clinical and Experimental Medicine, University of Sassari, 07100 Sassari, Italy; ^2^Endocrinology Unit, Azienda Ospedaliero-Universitaria, 07100 Sassari, Italy; ^3^Baylor College of Medicine, Houston, TX 77030, USA

## Abstract

*Background*. Intensive transfusion schedule and iron-chelating therapy prolonged and improved quality of life in patients with *β*-thalassemia (*β*-T) major. However, this led to an increased risk of developing impaired glucose tolerance or diabetes. In this study we analyzed variables associated with the occurrence of impaired glucose tolerance or diabetes in patients with *β*-T major.* Methods*. 388 Sardinian patients were included. Age, gender, duration of chelation therapy, body mass index, and markers of pancreatic and extrapancreatic autoimmunity were analyzed.* Results*. Multiple logistic regression analysis showed that anti-thyroid peroxidase (TPO) antibodies (Ab) (OR = 3.36; *p* = 0.008) and male gender (OR = 1.98; *p* = 0.025) were significantly associated with glucose impairment, while the other variables were not. Ferritin levels were significantly higher in TPOAb positive compared to TPOAb negative patients (4870 ± 1665 *μ*g/L versus 2922 ± 2773 *μ*g/L; *p* < 0.0001).* Conclusions*. In patients with *β*-T major a progressive damage of insulin-producing cells due to secondary hemosiderosis appears to be the most reasonable mechanism associated with glucose metabolism disorders. The findings need to be confirmed with additional well designed studies to address the question of whether TPOAb may have a role in the management of these patients.

## 1. Introduction

Beta-thalassemia (*β*-T) is one of the most common heritable disorders, due to mutations in the gene coding for the hemoglobin *β*-chain [1, 2]. It is widespread, especially in tropical and subtropical areas of the world (Middle East, sub-Saharan Africa, and South East Asia), as well as in the Mediterranean populations [3]. In homozygotes or compound heterozygotes, the disease is characterized clinically by severe anemia (Cooley disease) requiring intensive blood transfusion treatment [4]. In the past three decades this treatment significantly extended patients' lifespan and improved the quality of their life. However, frequent transfusions result in iron deposition in the majority of tissues producing organ injury [5]. Early chelation therapy offers some protection against transfusional iron overload, but the frequency of complications related to secondary hemosiderosis is still considerable. Progressive glucose intolerance or overt diabetes is the main metabolic complication in a significant proportion of patients with *β*-T major and contributes to overall morbidity [6–8]. However, the pathogenetic mechanisms leading to diabetes are still poorly understood, and most authors currently believe that iron deposition in the pancreatic *β*-cells is the leading cause [6, 9, 10]. This hypothesis is supported by the increased incidence of diabetes observed in idiopathic hemochromatosis [10] and in posttransfusional iron overload [6]. This opinion is further reinforced by the evidence of increased fasting proinsulin levels and proinsulin-to-insulin ratio in patients with thalassemia, indicating early beta-cell damage due to siderosis [11]. This finding is independent of the glucose tolerance status and often is directly correlated with serum markers of long term iron toxicity such as serum ferritin, liver iron, serum aspartate aminotransferase, and the duration of the transfusion treatment [12]. Moreover, liver disorders and genetic influence seem to be additional predisposing factors [13]. In particular insulin resistance, due to liver damage, has been claimed to exert a pivotal role in the development of this complication [14]. A previous study in a small sample of patients with *β*-T major investigated the relative importance of autoimmunity and insulin resistance on the risk of developing impaired glucose tolerance or diabetes [15]. However, among clinical and biochemical variables analyzed, the authors did not take into account extrapancreatic autoimmune markers. Therefore, the possible role of autoimmunity is not yet fully elucidated in this hematologic disorder. Based on literature data, we performed this study in order to evaluate clinical and immunological variables associated with impaired glucose metabolism or diabetes in a large cohort of transfusion-dependent patients with *β*-T major from Sardinia, Italy.

## 2. Patients and Methods

This was a cross-sectional study based on examination of clinical records of 388 patients with a diagnosis of *β*-T, including 340 with thalassemia major and 48 with thalassemia intermedia, treated with regular (every 2-3 weeks), long term blood transfusions and chelating agents (desferoxamine or a combination of desferoxamine and another iron-selective chelating molecule). However, dosage of chelating treatment was not available. A total of 388 patients (212 females; mean age: 32.9 ± 6.7 years) were recruited at the Microcytemic Center of Sassari, Italy, in the last three decades. Diagnostic criteria for IGT and diabetes were those recommended by the American Diabetes Association (ADA) [16]. Briefly, patients underwent the standard 75 g oral glucose tolerance test (OGTT). Diabetes was defined as having a fasting plasma glucose ≥7.0 mmol/L and IGT as a postprandial glucose level at 120 min after the glucose load (2 hPG) ≥7.8 mmol/L but <11.1 mmol/L. For all patients included in the study demographic data, biochemical tests such as fasting blood glucose and OGTT, serum ferritin, liver function tests, and hepatitis B and C markers were retrieved from the clinical records. Informed consent was obtained from all patients included in the study and the study was approved by the local Ethical Committee.

### 2.1. Antibody Assay

To evaluate the presence of pancreatic autoimmune response, individuals were tested for glutamic acid decarboxylase 65 autoantibodies (GAD65Ab) by using a highly accurate radioligand binding assay [17]. Briefly, ^35^S-methionine-labeled GAD65 was produced by in vitro coupled transcription/translation with SP6 RNA polymerase and nuclease-treated rabbit reticulocyte lysate (Promega, Madison, WI, USA) as previously described [18]. Then, 2.5 *μ*L of sample was added to 60 *μ*L of ^35^S-labeled GAD65. In an immunoprecipitation buffer (IMP buffer, 150 mM NaCl, 20 mM Tris, 0.15% Tween 20, 10 mM benzamidine, 0.1% aprotinin, and 0.1% BSA, pH 7.4), the serum samples, at a final serum dilution of 1 : 25, were incubated overnight at 4°C in duplicate. Labeled antibody-bound GAD65 antigen was separated from free antigen after 45 minutes' incubation at 4°C in 96-well nitrocellulose microtiter plates with Protein A-Sepharose (Zymed, San Francisco, CA, USA) diluted to 40% with IMP buffer. After eight washes and drying, scintillation fluid was added and radioactivity was counted in a TopCount NXT (Packard). The interassay percent CV for the positive control sample was 15% for GAD65Ab. The assay showed 70% sensitivity and 98% specificity for GAD65Ab [18]. GAD65Ab positivity was expressed according to the 99th percentile cut-off, although a 95th percentile cut-off was also considered in multivariate analysis to include lower titers. Additional markers of organ-specific autoimmunity were anti-thyroid peroxidase (TPOAb) and antitransglutaminase antibodies (tTGAb) both evaluated in all patients. The TPOAb were measured by immunoradiometric assays using commercially available kits (Immunotech, Prague, Czech Republic [normal range 0–12 IU/mL]; DiaSorin, Saluggia, Italy [reference range 0–50 U/mL]), whereas tTGAb IgA was measured by means of ELISA commercial procedure provided by Eurospital (Trieste, Italy) [17]. Patients who resulted positive underwent a complete thyroid evaluation including physical examination, thyroid ultrasound scan, and the assessment of thyroid-stimulating hormone (TSH) and serum free T4 (FT4) and free T3 (FT3) levels.

### 2.2. Statistical Analysis

Variables positively associated with glucose alteration (impaired glucose tolerance or diabetes) were detected by performing a multiple logistic regression analysis taking the presence (“1”) or absence (“0”) of glucose alterations as dependent variable and the clinical and immunological parameters of *β*-T patients as independent variables. The titers of GAD65Ab were expressed as “high” (above 99th percentile) and “low” (below 99th percentiles) although in the multivariate analysis the GAD65Ab were categorized according to the 95th percentile to take into account also subthreshold values that may be associated with mild damage. TPOAb and tTGAb were coded into binary variables by giving “0” or “1” for titers within or exceeding normal values, according to the manufacturer's specified range. Differences in mean values were tested with two-tailed Student's *t*-test. Odds ratios (ORs) and their 95% confidence intervals (CI) were calculated as the relative amount of increase of odds from one-unit change in the independent variables, after controlling for the confounding effect of all covariates. Cox and Snell “pseudo”* R*-squared was used to assess model fit. A *p* value lower than 0.05 was considered to be statistically significant for all calculations. The analysis was carried out by using SPSS Enter procedure (SPSS statistical software, v. 16.0, Chicago, USA).

## 3. Results

The clinical and immunological characteristics of the 388 *β*-T major patients are reported in Table 1. The proportion of patients with impaired glucose tolerance (IGT) was 8.5% (33 out of 388) and with diabetes was 16.5% (64 out of 388). Among the 48 patients with thalassemia intermedia 5 (10.4%) had overt diabetes and only one (2.0%) had IGT. Patients with diabetes were slightly but significantly (*p* = 0.015) older than patients with normal glucose tolerance (NGT) or IGT. Gender ratio and BMI did not differ across groups (*p* = 0.242 and *p* = 0.298, resp.). Surprisingly, the duration of chelation therapy was about 2 years longer, on average, in patients with diabetes compared to the other groups (26.5 versus 24.3 years; *p* = 0.017). The frequency of GAD65Ab positivity above the threshold of 99th percentile among *β*-T patients with NGT was 0.3%, similar to the frequency in the general population, and was not significantly different from *β*-T patients with IGT or diabetes (zero and 3%, resp.; *p* = 0.094).

Forty-six patients out of 388 (12%) showed positivity for TPOAb (26 with normal glucose tolerance, 2 with IGT, and 18 with overt diabetes). The frequency of positivity for TPOAb was increased threefold in patients with diabetes compared to NGT patients (28.1% versus 9.0%, *p* < 0.0001) whereas the frequency of positivity for anti-tTGAb was negligible in all patient subgroups. Among the 46 patients who were TPOAb positive the number of those displaying increased TSH concentration (>5.0 *μ*IU/mL) was 19 out of 26 with NGT (73%), 1 out of 2 with IGT (50%), and 10 out of 18 with overt diabetes (56%); in addition, 7 NGT patients and 6 diabetic patients had also low FT4 and/or FT3. Ultrasonography, when available, showed mostly nonspecific findings, such as dishomogeneity and reduced thyroid diameter.

Serum ferritin levels were significantly higher in patients with TPOAb positivity than in those who were TPOAb negative (4870 ± 1665 versus 2922 ± 2773*μ*g/L; *p* < 0.0001).

The prevalence of chronic infection with hepatitis C virus was available only in 210 out of 388 patients tested and was found higher in IGT/diabetic than in nondiabetic subjects (64% versus 23%, resp.; *p* < 0.0001). Multiple logistic regression analysis is reported in Table 2. Among the variables included in the model only male gender (OR: 1.98, 95% CI = 1.09–3.60) and positivity for anti-thyroid peroxidase antibodies (OR: 3.37, 95% CI = 1.38–8.24) were significantly associated with glucose metabolism disorders. Cox and Snell “pseudo”* R*-squared was equal to 0.066.

## 4. Discussion

Intensive transfusions and iron chelation therapy have prolonged and improved the quality of life of patients with *β*-T major. However, along with life extension the risk of developing endocrine complications is increased, particularly diabetes mellitus whose prevalence in *β*-T patients was reported in the range of 2.3 to 24% [19, 20]. Most studies attributed the onset of diabetes to the toxic effects of chronic iron overload on *β*-cells [5–10], while relatively fewer studies have focused on the possible contribution of concomitant autoimmune mechanisms [15]. This problem is particularly evident in the Sardinian population considering its high incidence of both *β*-T and autoimmune diabetes [3]. A casual association of the two conditions cannot be completely excluded. In this retrospective study GAD65Ab, by using a home-made high-sensitivity assay, were measured in a large cohort of patients diagnosed with *β*-T major in one tertiary level hospital in order to assess the potential autoimmune component of diabetes. In the cohort studied the overall frequency of glucose metabolism impairment was 25% of which 16.5% was due to overt diabetes and 8.5% was due to OGTT-ascertained impaired glucose tolerance. These findings are comparable to that of other studies [8, 9, 11–14, 21] and are far below the prevalence reported recently in a UK cohort [13]. The frequency of positivity for GAD65Ab, defined according to a 99th percentile cut-off, in thalassemic patients with NGT or IGT was not different from the frequency in the general population. In patients with overt diabetes the frequency of GAD65Ab positivity was slightly increased (2 patients out of 64, 3.1%) but the titers were far below those normally found in other forms of autoimmune diabetes such as type 1 diabetes and latent autoimmune diabetes in adults [22]. This suggests that the presence of antibodies against *β*-cell in thalassemia patients is likely to be a transient reaction triggered by increased exposure to antigen released by *β*-cells as a result of progressive iron overload. This hypothesis is supported by recent studies that have linked the magnitude of pancreatic iron overload, as measured by magnetic resonance imaging, with the risk of developing glucose impairment [20]. Besides, already in the early study of Monge et al., *β*-cells autoimmunity was found limited only to anti-islet cell antibodies which are less specific and mostly reflect a reactive process, whereas anti-GAD65 antibodies were not detectable [15]. However, at present a role played by genetic factors associated with increased risk of autoimmune diabetes, some of which can selectively enhance the level of circulating GAD65Ab triggered by nonimmune mechanisms, cannot be definitely excluded. Unfortunately in our study we were not able to test this hypothesis because data about genotyping were not available. One more limitation of this study is that the role of insulin resistance was not addressed specifically. However statistical analysis did not reveal any significant association of BMI with glucose tolerance disorders, although the limited BMI variability given by the young age of participants might have partially affected the results. According to previous observation by Fung et al. [23] a normal weight was also detected in our diabetic patients with *β*-T. Likely, the primary illness (*β*-T) may have a stronger influence on body composition than related comorbidities. The same consideration applies to the duration of iron chelation: in our dataset the variability was too limited to make any meaningful inference possible.

The strong association between the occurrence of diabetes and positivity for TPOAb is the main result of the present study. The percentage of patients with TPOAb positivity who also had subclinical hypothyroidism was substantially similar in NGT and diabetic groups. This makes it highly likely that the increased level of TPOAb is a marker of iron-mediated tissue damage rather than being entirely due to a primary autoimmune process of the thyroid gland. The finding of a significant increase in ferritin levels in positive TPOAb patients compared to negative ones is consistent with this interpretation.

In this study we have found that male gender has a strong association with diabetes, a result for which no obvious explanation is currently available, apart from the consideration that the same trend has been observed in other iron loading conditions [24]. In hereditary hemochromatosis signs of iron overload are much more common in men [25, 26] and the mean ferritin levels are lower in women (54 versus 344 microgr/L) [24]. This is usually attributed to the protective effect of chronic blood loss in women owing to menstruation and deliveries. Similar findings have been reported also in animal models [27]. However most beta-thalassemia female patients in our dataset displayed amenorrhea due to pituitary and gonadal damage caused by secondary hemochromatosis, what makes such a mechanism less plausible. Moreover organ-specific dysfunctions due to iron overload in beta-thalassemia such as hypogonadism and hypothalamic-pituitary axis impairment are more evident in men [28]. At the moment no hypothesis of this gender difference is supported by evidence and further research in this field is necessary to clarify our results.

The analysis carried out in this study has certain strengths and limitations: (i) organ-specific autoimmunity was investigated in a quite high number of patients from a population ethnically and genetically very homogeneous thus minimizing potential confounders and (ii) the antibody titers were measured by means of a home-made method whose accuracy was well documented and with a sensitivity far superior than most of that provided by commercial kits; (iii) however pancreatic autoimmunity was assessed only by means of GAD65Ab while other islet autoantibodies (such as IA2-Ab and ZnT8-Ab) were not included in the analysis, which may have underestimated the true prevalence of beta-cell autoimmunity; (iv) the lack of longitudinal data as well as the possibility of assessing the liver iron content hampered the evaluation of the effect of chelation therapy on iron overload; (v) in addition, markers of glycemic control were not directly available and a potential condition of prediabetes status was derived only from the information available in the clinical record the oldest of which dated back to thirty years ago. This limitation was not considered of major importance since the study focused essentially on the hypothetical relationship between autoimmunity and further development of diabetes.

## 5. Conclusions

In patients with *β*-T a progressive damage of insulin-producing cells due to secondary hemosiderosis still seems to be the most reasonable mechanism explaining the onset of glucose impairment, while additional loss of insulin-secreting cells by autoimmune processes, although cannot be definitely ruled out, is far less probable. The coexistence of a positivity of anti-thyroid antibodies, acting as indirect markers of iron overload in the thyroid rather than expression of ongoing autoimmune aggression in this tissue, is highly related to the presence of diabetes. Although our findings support an association between anti-thyroid antibodies and the presence of diabetes in patients with *β*-T, this relationship should be taken cautiously. More well designed studies, including imaging, are needed to confirm our observations before considering the TPOAb a useful tool in clinical practice.

## Figures and Tables

**Table 1:** Characteristics of 388 thalassemic patients according to the presence of disorders of glucose metabolism.

Variable	NGT	IGT	Diabetes	*p* value^*∗*^
Number of patients	291 (75%)	33 (8.5%)	64 (16.5%)	—
Age (years)	31.1 ± 7.4	31.9 ± 5.3	33.9 ± 5.2	**0.015**
Gender (F : M)	164 : 127	14 : 19	34 : 30	0.242^#^
Chelation duration (years)	23.9 ± 6.9	24.3 ± 5.5	26.5 ± 5.3	**0.017**
BMI (kg/m^2^)	21.4 ± 3.1	21.3 ± 1.9	22.1 ± 2.4	0.298
GAD65Ab > 95th percentile	10.6%	12.1%	18.8%	0.192
GAD65Ab > 99th percentile	0.3%	0.0%	3.1%^§^	0.094
TPOAb+	9.0%	6.1%	28.1%	**<0.0001**
tTG+	1.7%	0.0%	1.6%	0.748

NGT, normal glucose tolerance; IGT, impaired glucose tolerance.

^*∗*^One-way ANOVA; ^#^cumulating IGT and diabetes.

^§^In the two type 1 diabetes patients antibody titers were, respectively, GAD65Ab 0.41 mmol/L and 0.33 mmol/L; TPOAb = zero and 25 IU/mL; and tTGAb was negative in both cases.

**Table 2:** Multiple logistic regression analysis to test the association between covariates and the probability of developing alteration of glucose metabolism (IGT or overt diabetes).

Covariates	Beta	Standard error	Significance	ORs	95% CI for ORs
Age (years)	−0.018	0.039	0.641	0.982	(0.909–1.061)
Male gender	0.683	0.306	**0.025**	1.981	(1.088–3.605)
Chelation duration (years)	0.022	0.038	0.565	1.022	(0.949–1.101)
BMI (kg/m^2^)	0.040	0.053	0.457	1.040	(0.937–1.154)
GAD65Ab > 95th perc	0.481	0.461	0.296	1.618	(0.656–3.993)
TPOAb+	1.215	0.457	**0.008**	3.369	(1.377–8.243)
Constant	−0.701	1.505	0.641	—	—
